# Comparative genomics of the type VI secretion systems of *Pantoea *and *Erwinia *species reveals the presence of putative effector islands that may be translocated by the VgrG and Hcp proteins

**DOI:** 10.1186/1471-2164-12-576

**Published:** 2011-11-24

**Authors:** Pieter De Maayer, Stephanus N Venter, Tim Kamber, Brion Duffy, Teresa A Coutinho, Theo HM Smits

**Affiliations:** 1 Forestry and Agricultural Biotechnology Institute, University of Pretoria, South Africa; 2 Agroscope Changins-Wädenswil ACW, Division of Plant Protection, Wädenswil, Switzerland

## Abstract

**Background:**

The Type VI secretion apparatus is assembled by a conserved set of proteins encoded within a distinct locus. The putative effector proteins Hcp and VgrG are also encoded within these loci. We have identified numerous distinct Type VI secretion system (T6SS) loci in the genomes of several ecologically diverse *Pantoea *and *Erwinia *species and detected the presence of putative effector islands associated with the *hcp *and *vgrG *genes.

**Results:**

Between two and four T6SS loci occur among the *Pantoea *and *Erwinia *species. While two of the loci (T6SS-1 and T6SS-2) are well conserved among the various strains, the third (T6SS-3) locus is not universally distributed. Additional orthologous loci are present in *Pantoea *sp. aB-valens and *Erwinia billingiae *Eb661. Comparative analysis of the T6SS-1 and T6SS-3 loci showed non-conserved islands associated with the *vgrG *and *hcp*, and *vgrG *genes, respectively. These regions had a G+C content far lower than the conserved portions of the loci. Many of the proteins encoded within the *hcp *and *vgrG *islands carry conserved domains, which suggests they may serve as effector proteins for the T6SS. A number of the proteins also show homology to the C-terminal extensions of evolved VgrG proteins.

**Conclusions:**

Extensive diversity was observed in the number and content of the T6SS loci among the *Pantoea *and *Erwinia *species. Genomic islands could be observed within some of T6SS loci, which are associated with the *hcp *and *vgrG *proteins and carry putative effector domain proteins. We propose new hypotheses concerning a role for these islands in the acquisition of T6SS effectors and the development of novel evolved VgrG and Hcp proteins.

## Background

The Type VI secretion system (T6SS) was first identified in the human pathogens *Pseudomonas aeruginosa *and *Vibrio cholerae *and was shown in the latter to function as an injectisome for the delivery of pathogenicity effector proteins into the host cell [1,2]. T6SS have since been identified in genome sequences of many Gram negative bacteria, including those of several animal and plant pathogens, but also in symbiotic and free-living bacteria [3]. This has led to the speculation that the T6SS is involved in other non-pathogenic functions including inter-bacterial communication, regulating biofilm formation and environmental stress response [4,5]. In the animal pathogens *Salmonella enterica *subsp. *enterica *serovar Typhimurium and *Helicobacter hepaticus *mutational analysis indicated that the T6SS suppresses virulence and promotes replication in host cells, thus contributing to long-term colonization [6,7]. In other bacteria, including the animal pathogen *Yersinia pestis *and the phytopathogen *Pectobacterium atrosepticum*, this increased proliferation has been hypothesized to result in increased fitness and, subsequently, increased virulence [8,9]. The T6SS of the plant symbiont *Rhizobium leguminosarum *has been linked to host-specificity, where a T6SS deletion mutant of a strain restricted to *Trifolium subterraneum *(clover cv. Woogenellup) acquired the ability to form nitrogen-fixing nodules on the non-host *Pisum sativum *(pea) [10]. Several authors have identified an antibacterial role for the T6SS. A secreted effector, Tse2, in *P. aeruginosa *has been shown to have toxic effects on other bacteria [11]. Furthermore, the T6SS effectors Tse1 and Tse3 represent lytic enzymes that degrade peptidoglycan in the cell walls of closely related Gram negative bacteria [12]. Similarly, bactericidal functions have been ascribed for T6SSs in *V. cholerae *[13] and *S. enterica *[14]. The availability of more genome sequences and more experimental data will facilitate a greater understanding of new and known functions of the T6SS. Several bacteria encode multiple T6SS loci on their genomes. For example, the genomes of *P. aeruginosa*, *Y. pestis *and *Burkholderia pseudomallei *encode three, four and six T6SS loci, respectively [3]. These seemingly non-paralogous loci, together with the divergent functions of the T6SS suggests that this secretory system could influence a variety of interactions within a single species, with various hosts, and/or with other bacteria occupying the same niche [3].

While the biological roles of the T6SS in many Gram negative bacteria still need to be elucidated, other aspects of this secretory system, including evolutionary, genetic, structural and regulatory facets are becoming better understood. T6SS loci are generally comprised of 15-25 genes and include a core of 16 conserved proteins, which assemble the T6SS apparatus in the cell membrane [15]. These conserved proteins include IcmF (COG3523) and DotU (COG3455), which have homology to proteins associated with the type IV secretion system and are thought to stabilize the T6SS apparatus in the cytoplasmic membrane [15]. An AAA+ ATPase, ClpV (COG0542) may energize the assembly of the apparatus. Other conserved proteins include the DUF770 (COG3516) and DUF877 (COG3517) proteins, which are predicted to function as chaperones and the COG3521 outer membrane lipoprotein [15]. The outer component of the T6SS apparatus is comprised of two proteins, VgrG (COG3501) and Hcp (COG3157), which have also been identified as secreted effectors of the T6SS [16]. These proteins resemble both structurally and genetically the components of the T4 bacteriophage tail spike, suggesting that the VgrG-Hcp combination forms a similar membrane penetrating structure and are evolutionarily related [16,17]. Hcp proteins form hexameric rings that are conceivably stacked to form a tube penetrating the outer membrane through which proteins are transported into the extracellular space [18]. The VgrG protein is postulated to puncture the outer cell membrane to allow extrusion of the Hcp tube and subsequently breach the host cell membrane [16].

VgrG proteins have been demonstrated to be secreted by the T6SS and are themselves required for the secretion of other effector proteins, such as the Tse3 effector in *P. aeruginosa *[19]. However, a VgrG protein in this organism has been shown to be secreted in a T6SS-independent manner [19]. This indicates that the VgrG proteins are distinct and may perform diverse functions. Some VgrG proteins have been found to carry C-terminal extensions, which carry conserved domains of various predicted functions. The N-terminal of these "evolved VgrGs" may serve in assembly of the T6SS machinery, while the C-terminal portion functions as effector [16]. One such evolved VgrG in *V. cholerae *(VgrG-1) carries an actin-binding domain in its C-terminal extension which has been shown to be involved in host cell toxicity [20]. The C-terminal extension of a VgrG in *S. enterica *subsp. *arizonae *(IIIa) carries an S-type pyocin domain which has been proposed to function in bacterial cell killing [14]. Over 500 VgrG orthologs have been identified in bacterial genome sequences with many of these representing evolved VgrGs. In a number of organisms for which genome sequences are available, additional Hcp and VgrG paralogs with a function in T6SS, termed "orphan" Hcps and VgrGs are encoded by genes located on the genome separately from the T6SS loci [14,21].

The genera *Pantoea *and *Erwinia *include ecologically diverse species, including the plant pathogens *Erwinia amylovora *and *Pantoea stewartii *subsp. *stewartii*, which are linked to devastating diseases of rosaceous plants and maize, respectively [22,23]. The opportunistic plant pathogen *Pantoea ananatis *causes disease on a broad range of host plants and has been associated with human bacteremia [24]. Several *Erwinia *species, including *Erwinia billingiae *and *Erwinia tasmaniensis*, are found as part of the normal epiphytic microflora of plants [25]. Strains of *Pantoea agglomerans *and *Pantoea vagans *have recently received attention as potent biological control agents [26-28]. Strains of both *Pantoea *and *Erwinia *have also been isolated from insects [29,30]. The complete and draft genomes of several *Pantoea *and *Erwinia *species have recently been sequenced, which provide an extensive base to identify genomic differences that may be linked to the divergent biological and ecological phenotypes of these species. We used the genome sequences to identify the T6SS in these organisms. An in depth analysis was undertaken which showed that not all T6SS loci are universal to the sequenced *Pantoea *and *Erwinia *species and that there are notable anomalies between some of the loci that are conserved among the compared organisms. We show that these differences occur in putative genomic islands associated with the Hcp and VgrG proteins and postulate that these islands represent regions of rapid and extensive evolution, which may drive functional diversification of the T6SS.

## Results and Discussion

### Several orthologous T6SS loci occur in *Pantoea *and *Erwinia *species

**Figure 1:**
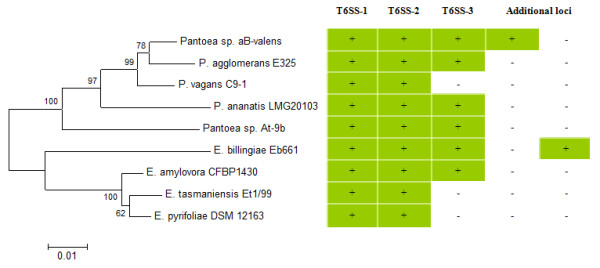
**Distribution of T6SS loci among *Pantoea *and *Erwinia *species**. The presence (+) or absence (-) of ortholgous T6SS loci for the various *Pantoea *and *Erwinia *strains are shown. A neighbor-joining tree (bootstrap n = 1,000) based on a ClustalW alignment of GyrB amino acid sequences indicates the phylogenetic relationship of the strains.

By means of baiting with the conserved proteins from several characterized T6SS in other bacteria, between two and four T6SS loci were identified in the complete or draft genome sequences of five *Pantoea *and four *Erwinia *strains (Figure 1). These strains were the *Eucalyptus *pathogen *P. ananatis *LMG 20103 [31], the biological control strains *P. vagans *C9-1 [32] and *P. agglomerans *E325 (Smits and Duffy, unpublished results), the insect-associated strains *Pantoea *sp. aB-valens [29] and *Pantoea *sp. At-9b [30] which represent novel species of the genus *Pantoea*, the fire blight pathogens *E. amylovora *CFBP 1430 [22] and *E. pyrifoliae *DSM 12163^T ^[33] and the apple epiphytes *E. tasmaniensis *Et1/99 [34] and *E. billingiae *Eb661 [25]. Two orthologous loci are found in all *Pantoea *and *Erwinia *strains, while a more restricted distribution could be observed for the T6SS-3 locus (Figure 1). Additional T6SS loci were identified in the genome sequences of *E. billingiae *Eb661 and *Pantoea *sp. aB-valens, respectively.

**Figure 2:**
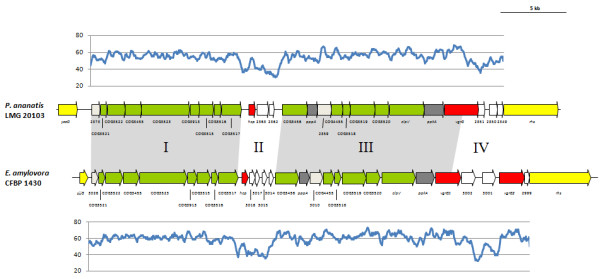
**The T6SS-1 loci of *P. ananatis *LMG 20103 and *E. amylovora *CFBP 1430**. The conserved regions (block I and III) are shaded in gray, while the non-conserved *hcp *and *vgrG *islands are not shaded. Genes encoding conserved domain proteins are represented by green arrows while the grey arrows indicate other genes conserved among the *Pantoea *and *Erwinia *T6SS-1 loci which were not identified as part of the conserved core described by Boyer et al. [15]. Red arrows represent the *hcp *and *vgrG *genes while genes not conserved among the *Pantoea *and *Erwinia *species are colored in white. Graphs show the G+C content (%) (window size = 50 bp, step = 10 bp) in the respective T6SS-1 loci.

**Figure 3:**
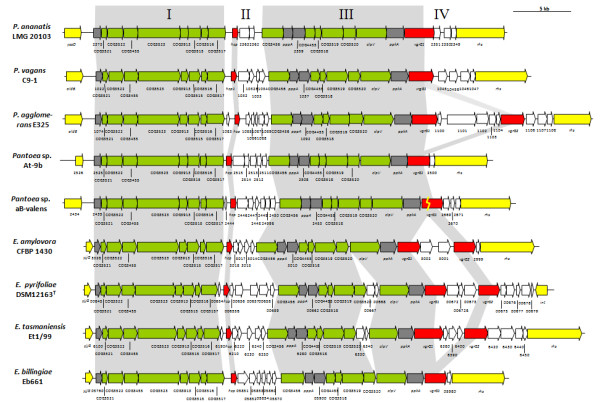
**The orthologous T6SS-1 loci in *Pantoea *and *Erwinia *species**. The conserved regions (block I and III) are shaded in gray, while the non-conserved *hcp *and *vgrG *islands are not shaded. Genes encoding conserved domain proteins identified by Boyer et al. [15] are represented by green arrows, and grey arrows indicate other genes conserved among the *Pantoea *and *Erwinia *T6SS-1 loci which were not identified as part of the conserved core described by Boyer et al. [15]. Red arrows represent the *hcp *and *vgrG *genes while genes not conserved among the *Pantoea *and *Erwinia *species are colored in white.

The T6SS-1 loci range in size from 28.8 and 40.0 kb and are built around a well-conserved and syntenous core which includes orthologs of all sixteen conserved T6SS proteins identified by Boyer et al. [15] (Figure 2 and 3). Additional genes encode a serine/threonine protein kinase (PpkA) and serine/threonine phosphatase (PppA). These proteins interact with the Fork Head-Associated (FHA) domain protein (COG3456) for the post-translational regulation of the T6SS [35]. In both *Pantoea *and *Erwinia *strains the conserved core T6SS proteins are arranged in two syntenous clusters (block I and III - Figure 2 and 3). These are separated by a variable region (block II), which is linked to the *hcp *gene (COG3157). A further variable region occurs next to the *vgrG *gene (block IV). In all sequenced *Pantoea *and *Erwinia *genomes, with the exception of *E. pyrifoliae *DSM 12163^T^, this variable region is flanked at the 3' end by a gene encoding a re-arrangement hot spot (*rhs*) element [36]. In *P. agglomerans *E325, *E. amylovora *CFBP 1430, *E. pyrifoliae *DSM 12163^T ^and *E. tasmaniensis *Et1/99, an additional copy of the *vgrG *gene occurs within this variable region. A non-conserved region harboring two hypothetical genes, which is not associated with a *vgrG *or *hcp *gene could be observed between COG3520 and *clpV *in *E. tasmaniensis *Et1/99 and *E. pyrifoliae *DSM 12163^T ^(Figure 3).

**Figure 4:**
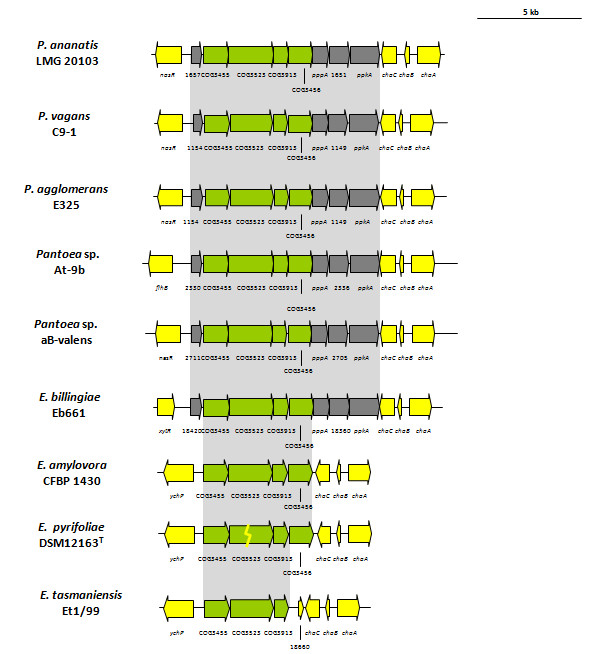
**The orthologous T6SS-2 loci in *Pantoea *and *Erwinia *species**. Genes encoding proteins with the conserved domains identified by Boyer et al. [15] are represented by green arrows while the grey arrows indicate other genes conserved among the *Pantoea *and *Erwinia *T6SS-2 loci which were not identified as part of the conserved core. White arrows represent the genes not conserved among the *Pantoea *and *Erwinia *species.

**Figure 5:**
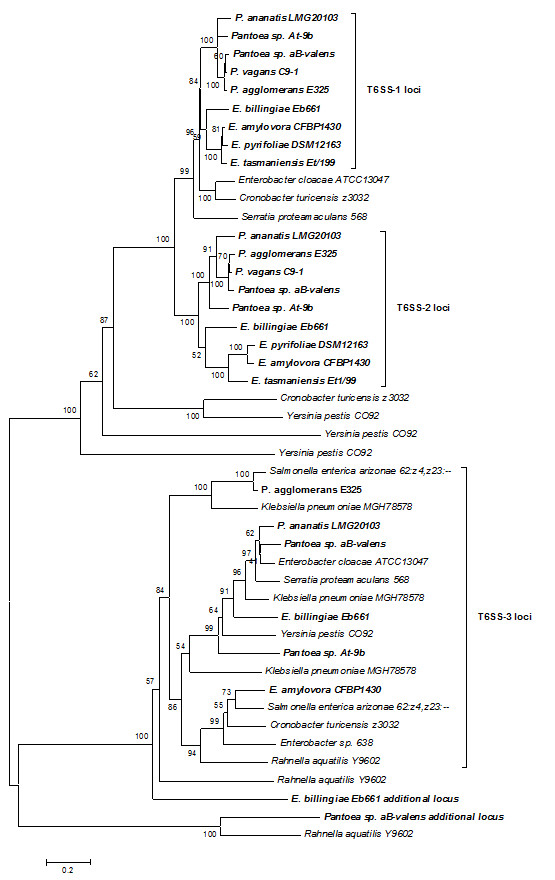
**Phylogenetic relationships between the T6SS loci among the *Pantoea and Erwinia *species**. A neighbor-joining tree (bootstrap n = 1,000; Poisson correction; Complete gap deletion) was constructed based on a ClustalW alignment of the IcmF (COG3523) amino acid sequences from the *Pantoea *and *Erwinia *T6SS loci and orthologous loci in several closely related *Enterobacteriaceae*.

The T6SS-2 loci of the *Pantoea *strains and *E. billingiae *Eb661 are structurally and genetically conserved and consist of eight syntenous genes which encode proteins that are highly conserved among the sequenced strains (Figure 4). Only four of these belong to conserved T6SS proteins outlined by Boyer et al. [15], suggesting the T6SS-2 loci encodes a partial and potentially non-functional T6SS. An average amino acid identity of 52% could be observed between the proteins encoded in the T6SS-2 locus and their respective paralogs in the T6SS-1 locus for each of the strains. This suggests that the T6SS-2 locus could have arisen through a partial duplication of the T6SS-1 locus. This is supported by phylogenetic analysis with the IcmF (COG3523) protein sequences (Figure 5), where the T6SS-2 loci branch from the T6SS-1 loci. The *E. amylovora *CFBP 1430 and *E. pyrifoliae *DSM12613^T ^T6SS-2 loci encode only four proteins, lacking the genes encoding the serine/threonine kinase and phosphatase required for posttranslational regulation, while an FHA domain (COG3456) protein is encoded in these loci. The *icmF *gene in the *E. pyrifoliae *DSM12613^T ^T6SS-2 locus is inactivated by a frameshift [33], while the *E. tasmaniensis *Et1/99 T6SS-2 locus is missing the FHA domain gene, indicating further gene loss in these T6SS loci (Figure 4).

**Figure 6:**
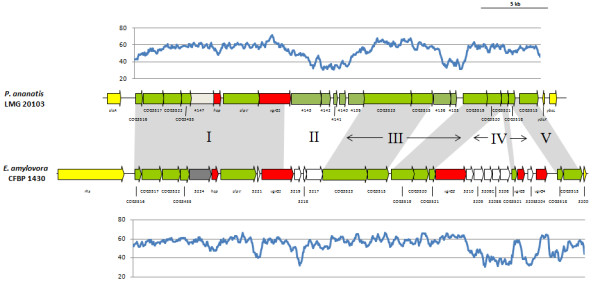
**The T6SS-3 loci of *P. ananatis *LMG 20103 and *E. amylovora *CFBP 1430**. The conserved regions (block I, III and V) are shaded in gray, while non-conserved regions are not shaded. Genes encoding conserved domain proteins identified by Boyer et al. [15] represented by green arrows while the grey arrow indicates a gene conserved among the *Pantoea *and *Erwinia *T6SS-3 loci which was not identified as part of the conserved core. Red arrows represent the *hcp *and *vgrG *genes while genes not conserved among the *Pantoea *and *Erwinia *species are colored in white. The graphs show the G+C content (%) (window size = 50 bp, step = 10 bp) in the respective T6SS-3 loci.

**Figure 7:**
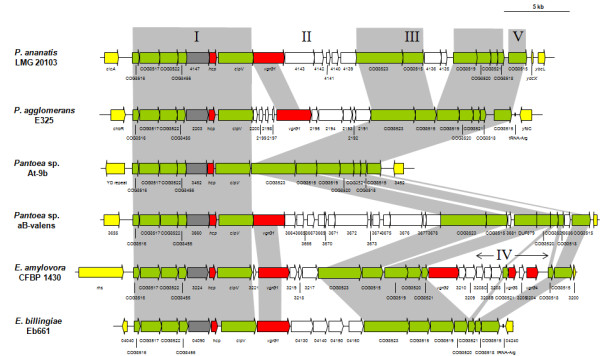
**The orthologous T6SS-3 loci in *Pantoea *and *Erwinia *species**. The conserved regions (block I, III and V) are shaded in gray, while non-conserved regions are not shaded. Genes encoding conserved core proteins identified by Boyer et al. [15] are represented by green arrows while the grey arrows indicate other genes conserved among the *Pantoea *and *Erwinia *T6SS-3 loci which were not identified as part of the conserved core. Red arrows represent the *hcp *and *vgrG *genes while genes not conserved among the *Pantoea *and *Erwinia *species are colored in white.

The *Pantoea *and *Erwinia *T6SS-3 loci are between 18.8 and 34.6 kb in size and encompass 19 to 32 protein coding sequences. In contrast to the T6SS-1 and -2 loci, the T6SS-3 locus is not universal to all *Pantoea *and *Erwinia *strains (Figure 1). The phylogeny based on the IcmF protein shows a greater evolutionary distance between the *Pantoea *and *Erwinia *T6SS-3 loci, which are interspersed with those of other *Enterobacteriaceae *(Figure 5), indicating that this locus may have been acquired through horizontal gene transfer. This can also be correlated with greater diversity in gene content and order between the various *Pantoea *and *Erwinia *T6SS-3 loci (Figure 6 and 7). Thirteen of the T6SS conserved core proteins identified by Boyer et al. [15] are encoded within the T6SS loci. Genes encoding orthologs of the COG3913, COG4455 and FHA domain (COG3456) proteins are absent. No PpkA or PppA orthologs are encoded in the T6SS-3 loci either, suggesting that this locus is not post-translationally regulated. As in the case of the T6SS-1 loci, syntenous blocks of core genes can be observed in the T6SS-3 loci (block I, III and V - Figure 6 and 7). Here, the *hcp *gene is included in a conserved block (Figure 6 and 7). The non-conserved regions in the T6SS-3 loci are generally associated with a *vgrG *gene. An additional complete and two partial *vgrG *genes can be found in the T6SS-3 locus of *E. amylovora *CFBP 1430. An additional non-conserved region, encoding two predicted proteins, PANA_4135 and PANA_4136 can be observed in the *P. ananatis *LMG 20103 T6SS-3 locus (Figure 7).

**Figure 8:**
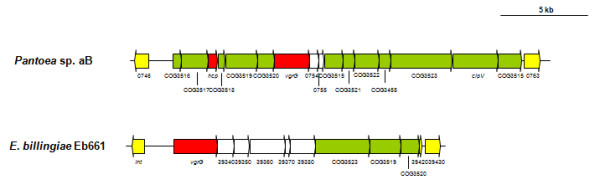
**Additional T6SS loci in *Pantoea *sp. aB-valens and *Erwinia billingiae *Eb661**. Genes encoding proteins with the conserved domains identified by Boyer et al. [15] are indicated by green arrows. Red arrows represent the *hcp *and *vgrG *genes while genes not conserved among the *Pantoea *and *Erwinia *species are colored in white.

A further T6SS locus was identified in *Pantoea *sp. aB-valens (Figure 8). This 19.6 kb locus encodes thirteen conserved core proteins. The IcmF phylogeny shows this T6SS locus to be distantly related to all other *Pantoea *and *Erwinia *T6SS loci. Similarly, an additional partial T6SS locus is encoded on the *E. billingiae *Eb661 genome, which contains four T6SS core proteins. A non-conserved region associated with the *vgrG *gene is likewise present. This *E. billingiae *Eb661 T6SS locus is flanked at both the 5' (EbC_39320 - phage integrase) and 3' end (EbC_393430-39600 - hypothetical phage genes) by genes of an integrated phage element. A phage origin has previously been hypothesized for the T6SS [17].

### Different VgrG proteins are encoded in the T6SS-1, T6SS-3 loci and additional T6SS loci

**Figure 9:**
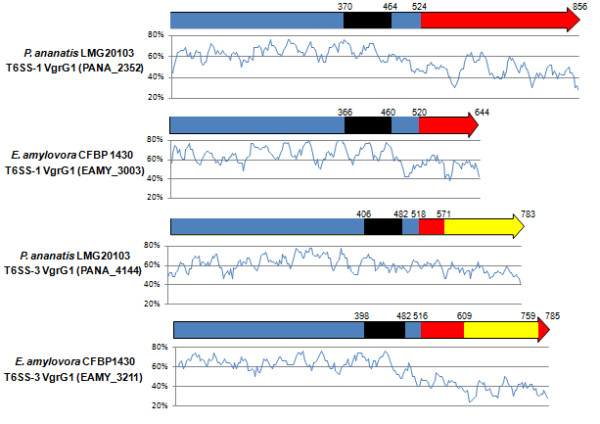
**Conserved and non-conserved domains in the VgrG proteins of *P. ananatis *LMG 20103 and *E. amylovora *CFBP 1430**. The conserved VgrG domains are colored in blue, Gp5 domains are in black, COG4253 domains in the T6SS-3 VgrG proteins in yellow and the non-conserved C-terminal extensions are colored in red. The amino acid locations of each domain are shown. The graphs show G+C contents (window size = 50 bp, step = 10 bp) in the respective *vgrG *genes.

**Figure 10:**
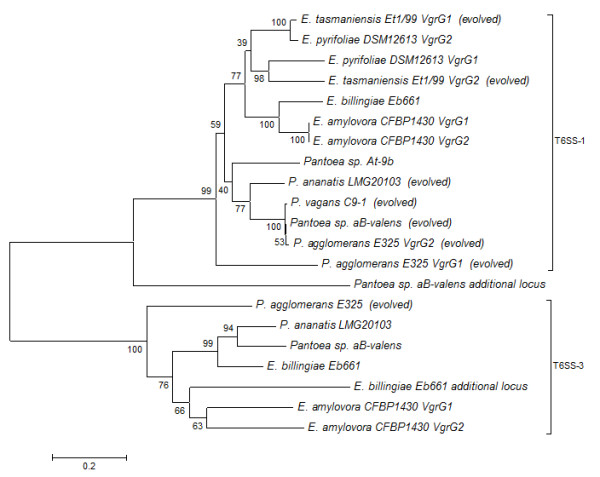
**Phylogeny of the *Pantoea *and *Erwinia *VgrG proteins**. Neighbor-joining tree showing the phylogenetic relationship between the VgrG proteins among the various *Pantoea and Erwinia *species. The tree (bootstrap n = 1,000; Poisson correction; Complete gap deletion) was constructed based on a ClustalW alignment of the VgrG amino acid sequences. "Evolved" VgrG proteins are indicated in brackets.

The amino acid sequences of all VgrG proteins encoded in the T6SS-1, T6SS-3 and additional T6SS loci in *E. billingiae *and *Pantoea *sp. aB-valens were analyzed for the presence of conserved and evolved domains. The N-terminal region of all T6SS-1, T6SS-3 and additional T6SS VgrG proteins is conserved among all *Pantoea *and *Erwinia *species and contains a conserved VgrG (TIGR3361 [37]) and phage Gp5 (pfam04717 [38]) domain (Figure 9). However, as has been observed in other bacteria, the VgrG proteins differ considerably in length between the various *Pantoea *and *Erwinia *species. This can be attributed to variable C-terminal regions, which suggest that they represent evolved VgrG proteins. Seven of the thirteen *Pantoea *and *Erwinia *T6SS-1 VgrG proteins contain such C-terminal extensions (Figure 10). Analysis of these C-terminal extensions showed that several of them contain conserved domains. The *P. agglomerans *E325 T6SS-1 VgrG2 protein (Pagg_1105) contains a peptidoglycan (PG)-binding domain (Pfam09374 [38] - Cdsearch score: 45) as well as COG3926 and COG5526 lysozyme domains [39,40], which were also found in the lytic bacteriophage φ8, suggesting a bacteriolytic function for this VgrG protein. A similar function can be expected for the *Pantoea *sp. aB-valens T6SS-1 VgrG (PANABDRAFT_2668) which contains a β-N-acetyl-glucosaminidase domain (COG4193 [40] - Cdsearch score: 58.5) in its C-terminal extension. The *E. tasmaniensis *Et1/99 T6SS-1 VgrG2 (ETA_06370) C-terminal extension shows structural homology to the *Streptomyces *sp. N174 chitosanase (1_chk_A - Hhpred score: 190) [41].

The C-terminal extensions of VgrG proteins encoded in the T6SS-3 loci of all *Pantoea *and *Erwinia *species contain a conserved domain of unknown function (COG4253), which is absent in the T6SS-1 loci VgrG proteins (Figure 9). This domain has also been identified flanking the N-terminal region of the *S. enterica *subsp. *arizonae *IIIa VgrG proteins, while it is not present in the VgrG proteins of other *Salmonella *serovars [14]. A role for the COG4253 domain, which links the N-terminal VgrG transporter and C-terminal extension, in modulating the function of the VgrG between secretion and virulence has been suggested [15]. However, with the exception of the *P. agglomerans *E325 T6SS-3 VgrG protein, which carries a C-terminal extension subsequent to the COG4253 region, no further C-terminal extensions are present in the *Pantoea *and *Erwinia *T6SS-3 VgrG proteins. An alternative function in host cell adhesion has also been suggested [16]. The latter function would imply that the COG4253-positive T6SS-3 and COG4253-negative T6SS-1 VgrG proteins could have different targets and serve different functions. A phylogeny based on the T6SS-1 and T6SS-3 VgrG proteins shows that they branch into two distinct clades suggesting distinct evolutionary backgrounds for these paralogous proteins (Figure 10). The VgrG protein found in the additional T6SS locus in *E. billingiae *Eb661 also contains a conserved COG4253 domain and clusters with the T6SS-3 VgrG proteins, while the additional *Pantoea *sp. aB-valens VgrG paralog clusters with the other VgrG proteins lacking this domain.

### Non-conserved *hcp*/*vgrG *regions represent islands within the T6SS loci

While the T6SS-1 and T6SS-3 loci share conserved and syntenous cores among the various *Pantoea *and *Erwinia *strains, considerable variability in the *vgrG *and *hcp *regions can be observed. The G+C deviation across these T6SS loci was determined. This showed that for the T6SS-1 and T6SS-3 loci, there is a much lower G+C content in the variable regions associated with the *hcp *and *vgrG *genes compared to the conserved core regions (Figures 2 and 4). In the T6SS-1 loci, the conserved core regions had an average G+C content of 60.17% across all strains, while the *hcp *regions (average G+C% = 42.30) and *vgrG *regions (average G+C% = 47.91) had a much lower G+C content. The substantial G+C deviations, variability in the gene content of the *hcp *and *vgrG *regions and the differential homology of proteins encoded in these regions to proteins in bacteria in other genera indicates that these regions represent variable islands within the T6SS loci. Similar G+C deviations could be observed for the *vgrG *regions in the additional *Pantoea *sp. aB-valens and *E. billingiae *Eb661 T6SS loci, which further supports that these regions serve as "hot spots" for rearrangement [36]. These hot spots are frequently associated with Rhs proteins which are capable of displacing its C-terminal tip and replacing it with a non-homologous alternative. By this means, the Rhs protein can drive the sequential insertion of heterogeneous C-terminal sequences into the hot spot [36]. As the additional *E. billingiae *Eb661 T6SS locus occurs within an integrated phage element, we postulate that transducing phages may play a role in the horizontal acquisition of non-conserved genes in the *vgrG *and *hcp *regions. Similar *vgrG*/*hcp *islands have also been identified in a number of *Pseudomonas *species [42]. These islands are associated with "orphan" *vgrG *and *hcp *paralogs separately located from the remainder of the T6SS loci. In contrast, this is the first observation of *hcp *and *vgrG *islands associated with the conserved core T6SS loci. Analysis of the T6SS loci of other bacteria (data not shown), however, shows that this phenomenon is not restricted to *Pantoea *and *Erwinia*.

### Hcp/VgrG islands harbor putative effector proteins

The proteins encoded in the variable *hcp *and *vgrG *islands in the T6SS-1 and T6SS-3 loci in the different *Pantoea *and *Erwinia *species were analyzed for sequence similarity and structural homology to known proteins and the presence of conserved domains. The majority of proteins encoded on the islands showed homology to proteins of unknown function. However, a number of island proteins share high sequence identity and contain conserved domains which suggest they may represent T6SS effectors with putative functions in host-microbe and inter-bacterial interactions (Additional File 1 Table S1).

The amino acid sequence of ETA_06430 encoded in the *E. tasmaniensis *Et1/99 T6SS-1 *vgrG *island shares homology with a phospholipase in *V. cholerae *HE48 (VCHE48_2681 - 40% aa identity) while that of E. *billingiae *Eb661 EbC_4130 encoded on the T6SS-3 *vgrG *island shows homology to a lipase/esterase in *Yersinia bercovieri *ATCC 43970 (Yberc0001_35290 -64% aa identity). The former also shows structural homology to a *P. aeruginosa *lipase (1ex9_A - Hhpred score: 115) while the latter shows structural homology to a lipase in *Penicillium expansum *(3g7n_A: Hhpred score: 157), supporting that these two proteins represent lipases. Similarly, EbC_39360 encoded in the additional T6SS of *E. billingiae *Eb661 shares weak structural homology with a lipase in *Archaeoglobus fulgidus *(2zyr_A - Hhpred score: 81.1). Lipases hydrolyze long-chain triglycerides into fatty acids and glycerol and have been shown to represent major virulence factor in both animal and plant pathogens [43,44]. Genes encoding lipases were also identified in the *vgrG *islands of *P. aeruginosa *and transcriptome analysis showed that their expression is co-regulated with that of the T6SS [42]. These lipases may therefore represent T6SS-secreted effectors in *P. aeruginosa *as well as the *Erwinia *species. Several genes in the *vgrG *regions of both *Pantoea *and *Erwinia *T6SS-1 and T6SS-3 loci may encode proteases. The *P. agglomerans *E325 Pagg2200 and *E. tasmaniensis *Et1/99 ETA_6240 proteins share 28% and 32% aa identity respectively with a putative zinc protease in *Acinetobacter baumanii *(AbauB_010100012633) and show weak structural homology to a zinc metalloproteases in *Geobacter sulfurreducens *(3c37_A - Hhpred score: 47.3 and 51.3, respectively). Furthermore, the *P. agglomerans *E325 Pagg_1085, *E. tasmaniensis *Et1/99 ETA_6210 and *E. amylovora *CFBP 1430 EAMY_3018 protein sequences show weak structural homology to the secreted cysteine protease stathopain (1cv8_A - Hhpred score: 29.8 34.2 and 34.2, respectively), which plays a role in skin infection by *Staphylococcus aureus *[45]. EbC_39340 and EbC_39350 localized in the *vgrG *island in the additional T6SS locus of *E. billingiae *Eb661 encode proteins with weak structural homology to the secreted *Clostridium perfringens *sialidase NanI (2bf6_A - Hhpred score: 46 and 44.3, respectively) which is involved in the removal of sialic acids from host glycoconjugates with an important role in bacterial nutrition and pathogenesis [46]. PANA_4143 associated with the T6SS-3 in *P. ananatis *LMG 20103, shares high sequence homology with the M23 peptidase of *Klebsiella *sp. 92-3 (HMPREF9538_05689: 78% aa identity) and extensive structural homology to the secreted chitinase G of *Streptomyces coelicolor *of (1chk_A - Hhpred score: 203). Chitinases degrade chitin, the carbohydrate polymer found in insect shells and the cell walls of fungi, suggesting the T6SS of *P. ananatis *LMG 20103 may secrete an effector with an antifungal or insecticidal function [47].

Two proteins, PANA_2363 and Pagg_2194, encoded in the T6SS-1 and T6SS-3 *hcp *islands of *P. ananatis *LMG20103 and *P. agglomerans *E325, respectively contain conserved peptidoglycan binding (Pfam01474 - PG_binding_1) and LysM (Pfam01476) domains which are typically associated with proteins involved in bacterial cell wall degradation. The latter also shows sequence homology to a putative lytic enzyme in *Acinetobacter calcoaceticus *RUH2202 (HMPREF0012_02474 - 34% aa identity). Similarly, *E. pyrifoliae *DSM 12163^T ^Epyr_0675 shares sequence homology with a *Edwardsiella tarda *FL6-60 lysozyme (ETAF_ple052 - 32% aa identity) and also contains a conserved peptidoglycan binding domain (Pfam09374 - PG_binding_3) and a domain of unknown function (DUF847) frequently observed in lysozymes. The *E. billingiae *Eb661 EbC_05851 protein also shares sequence homology with the *Pseudomonas *phage PaP1 endolysin (PaP1_gp072 - 46% aa identity). The presence of domains conserved in bacterial cell wall degrading enzymes and the sequence homology to these enzymes indicate that these proteins may play a similar role to the Tse1 and Tse3 lytic enzymes in *P. aeruginosa *in the degradation of the bacterial cell wall and may thus have a bactericidal function [12,48]. Furthermore, PANA_4136 encoded in the additional non-conserved region in the *P. ananatis *LMG 20103 T6SS-3 locus shows extensive sequence homology to S-pyocin domain containing proteins in *E. coli *3030-1 (EC30301_3278 - 58% aa identity) and *Yersinia pseudotuberculosis *IP 31758 (YpsIP31758_0897 - 53% aa identity) and shows weak structural homology to the colicin S4 of *Escherichia coli *(3few_X - Hhpred score: 45.5). Similarly, weak structural homology to colicin S4 can be observed for the T6SS *hcp *island *P. vagans *C9-1 Pvag_1032, *Pantoea *sp. At-9b Pat9b_2515 and *Pantoea *sp. aB-valens PANABDRAFT_2446 proteins (3few_X - Hhpred score: 60, 58.5 and 58.4, respectively). Colicins and pyocins are bacteriocins that are involved in killing closely related bacterial species [49]. The presence of bacteriocin-like proteins in the T6SS loci of these *Pantoea *species agrees with the finding of a potential function for the T6SS in antibiosis and competition [11,12].

### *Pantoea *and *Erwinia *VgrG and Hcp proteins may carry effector proteins encoded in the *vgrG *and *hcp *islands

Analysis of the G+C contents of the *Pantoea *and *Erwinia *T6SS-1 *vgrG *genes shows that the N-terminal regions, which contain the conserved VgrG and Gp5 domains, have an average G+C content of 63.68%, which is similar to the conserved core of the T6SS-1 loci. By contrast, the C-terminal extensions have an average G+C content of 46.59%. This is similar to the non-conserved *vgrG *island, suggesting that the C-terminal extensions form part of the *vgrG *islands. In the T6SS-3 loci, the conserved N-terminal region of the *vgrG *genes has an average G+C content of 59.01% while the *vgrG *islands have an average G+C content of 48.38%.

Some of the proteins encoded in the *vgrG *islands show sequence homology and contain conserved domains found in the C-terminal extensions of evolved VgrG proteins. The *E. pyrifoliae *DSM 12163^T ^T6SS-1 Epyr_00675 amino acid sequence shares 75% aa identity with the C-terminal region of the *P. agglomerans *E325 T6SS-1 evolved VgrG (Pagg_1105). The *P. ananatis *LMG 20103 T6SS-1 PANA_2363 and the C-terminal region of the evolved T6SS-1 VgrG of *P. agglomerans *E325 (Pagg_1105) also each contain a PG-binding domain and a LysM domain (Pfam01476) found in lytic enzymes, and show structural homology a cell wall degrading lysozyme in *Pseudomonas *phage Phikz (2kbh_A - Hhpred score: 128 and 44.5, respectively). BlastP analysis with the proteins encoded in the *vgrG *islands against the NCBI protein database showed that proteins encoded in the *vgrG *islands of several *Pantoea *and *Erwinia *species show substantial sequence identity to the C-terminal extensions in VgrG in bacteria belonging to other genera (Additional File 1 Table S1). The putative lytic enzymes encoded in the T6SS-1 *vgrG *islands of *P. ananatis *LMG 20103 (PANA_2363) and *E. billingiae *Eb661 (EbC_05851) share homology with the C-terminal extensions in the VgrG proteins of *V. cholerae *TMA21 (VCB_002278 - 41% aa identity) and *V. cholerae *CT 5369-63 (VIH_000452 - 49% aa identity), respectively. The putative zinc proteases encoded in the *P. agglomerans *E325 T6SS-3 *vgrG *island (Ppag_2200) and the *E. tasmaniensis *Et1/99 T6SS-1 *vgrG *island (ETA_06240) share 43 and 46% aa identity with the C-terminal extension of a *Burkholderia *sp. 383 VgrG protein (Bcep18194_C7612). Similarly, the putative *E. tasmaniensis *Et1/99 lipase (ETA_06430) shows 42% aa identity to the *Burkholderia glumae *BGR1 VgrG (Bglu_2g02560). The sequence homology and the presence of shared conserved domains between the C-terminal extensions of evolved VgrG proteins and putative effector proteins encoded in the *vgrG *islands, suggest that VgrG proteins may carry effector proteins encoded in the *vgrG *islands. The COG4253 conserved domain found at the C-terminal end of all the T6SS-3 VgrG proteins may be involved in the anchorage of the VgrG transporter to the effector proteins, thereby modulating the function of the VgrG protein as a structural component of the T6SS apparatus and the transport of effector proteins [15]. This domain is absent in the T6SS-1 loci, and the means by which effectors become associated with the VgrG transport region would need to be determined.

In the fish pathogen *Edwardsiella tarda*, the secreted effector protein EvpP has been shown to interact with the Hcp protein [50]. Furthermore, Hcp orthologs with C-terminal extensions have been identified in *S. enterica*, which may represent evolved Hcp proteins [14]. It is therefore plausible that, as is the case for the *Pantoea *and *Erwinia *VgrG proteins, the Hcp proteins may transport effector proteins encoded on the *hcp *islands. By this means, various effector proteins could be tagged to the VgrG and Hcp proteins, thereby forming different VgrG-effector and Hcp-effector combinations, which may perform different biological functions. The VgrG or Hcp proteins could transport bacteriocins or pyocins, which would allow the bacterium to target other bacteria competing for an ecological niche. A chitinase effector carried by the VgrG protein could play an antifungal or insecticidal role in the *Pantoea *and *Erwinia *species. These and other putative pathogenicity factors encoded by hypothetical proteins in the *hcp *and *vgrG *islands translocated by the VgrG and Hcp proteins may therefore enable *Pantoea *and *Erwinia *species to target a range of bacterial, invertebrate, vertebrate and or plant hosts.

## Conclusions

Comparative analysis of the genome sequences of several *Pantoea *and *Erwinia *species revealed that they encode between two and four T6SS loci. This suggests an important biological role for this secretion system in these two genera. Two of the T6SS loci, T6SS-1 and T6SS-2 are shared among all *Pantoea *and *Erwinia *strains, while orthologs of the third locus are only found in four of five *Pantoea *species and two of four *Erwinia *species, suggesting acquisition by horizontal gene transfer of this locus. *Pantoea *sp. aB-valens and *E. billingiae *Eb661 encode additional T6SS loci. Analysis of the T6SS-1, T6SS-3 and additional loci in *Pantoea *sp. aB-valens and *E. billingiae *Eb661 showed that while synteny is conserved among the *Pantoea *and *Erwinia *species for each locus, non-conserved regions could be observed associated with the *hcp *and *vgrG *genes. The G+C contents of these non-conserved regions differ substantially from the conserved portions of the loci, indicating horizontal acquisition of these regions separate from the rest of the T6SS loci. Several of the VgrG proteins encoded in the loci have a C-terminal extension and represent evolved VgrG proteins. These C-terminal extensions likewise have lower G+C contents than the remainder of the T6SS suggesting they form part of the *vgrG *islands.

Many of the proteins encoded in the *vgrG *and *hcp *islands carry conserved domains and show sequence and structural homology to proteins with various biological functions including antibiosis, fungal cell wall degradation and putative roles in animal and plant pathogenesis. We postulate that the *vgrG *and *hcp *islands may represent evolutionary hot spots for genes that encode effector proteins. Similar rearrangement hot spots have been observed in the regions adjacent to the *hcp *and *vgrG *genes in the T6SS loci of other bacteria [18,36], suggesting that this is a more widespread phenomenon. The sequence similarity and structural homology of some of these putative effector proteins to C-terminal extensions in characterized evolved VgrG proteins suggest that they may become tagged to the conserved core VgrG proteins which serve as transporters for these effectors, thereby forming new evolved VgrG proteins. Similarly, putative effectors encoded in the *hcp *islands may become associated with the Hcp proteins to form evolved Hcp proteins. We could therefore speak of "evolving" Hcp and VgrG proteins. By tagging various effector proteins different VgrG-effector and Hcp-effector combinations could be formed which may perform different biological functions. Thereby, the genomic islands associated with the Hcp and VgrG proteins could drive functional diversification of the T6SS, which may explain the plethora of biological roles described for this secretion system.

## Methods

### *In silico *identification of the T6SS loci in *Pantoea *and *Erwinia*

The T6SS loci in five *Pantoea *and four *Erwinia *species, for which complete or draft genome sequences are available, were identified: *Pantoea ananatis *LMG 20103 [31], *Pantoea vagans *C9-1 [32], *Pantoea agglomerans *E325 (Smits and Duffy, unpublished results), *Pantoea *sp. aB-valens [29], *Pantoea *sp. At-9b [30], *Erwinia amylovora *CFBP 1430 [22], *Erwinia pyrifoliae *DSM 12163^T ^[33], *Erwinia tasmaniensis *Et1/99 [35] and *Erwinia billingiae *Eb661 [25]. Identification of the T6SS loci was done by BlastP analysis [51] with the conserved core proteins identified by Boyer et al. [15] against local protein databases created for the *Pantoea *and *Erwinia *strains. Proteins neighboring *Pantoea *and *Erwinia *T6SS clusters were compared using BlastP against the NCBI protein database [52] to identify to full extent of the T6SS loci. Sequence manipulations were conducted with multiple subroutines of the LASERGENE package (DNASTAR, Madison, WI, USA).

### Phylogenetic analyses

Phylogenetic analyses were done using the procedures outlined by Bingle et al. [53]. A ClustalW alignment with default parameters was performed with the IcmF (COG3523) amino acid sequences, as this represents the only protein conserved among all *Pantoea *and *Erwinia *T6SS loci. A phylogenetic tree was constructed with the Molecular Evolutionary Genetics Analysis (MEGA) v. 5.0.3 software package [54], using the neighbor-joining method, with Poisson correction, complete gap deletion and bootstrapping (n = 1,000) parameters. The IcmF amino acid sequences from the T6SS loci of several closely related organisms as identified by BlastP analysis against the NCBI protein database [52] were included in the tree. This same procedure was employed to construct phylogenetic trees for Gyrase B (GyrB) and the VgrG proteins.

### Analyses of the *hcp*/*vgrG *islands

The average G+C contents for the conserved core regions and *hcp *and *vgrG *islands of the T6SS loci were determined using the Bioedit v.7.0.5.3 package [55]. The G+C content was determined using 50 bp windows in 10 bp steps. Similarly, the G+C contents for the *vgrG *genes were determined for the conserved N-terminal region, which included the conserved Vgr and Gp5 domains, and for the C-terminal extension which was considered as all nucleotides located at the 3' end of the Gp5 domain. Proteins encoded in the *vgrG *and *hcp *islands were identified using the FgenesB [56] and Orf finder [57] web servers. The amino acid sequences for the proteins encoded in the *hcp *and *vgrG *islands were analyzed for sequence identity by BlastP analysis against the NCBI protein database [51,52] the presence of conserved domains by Blast analysis against the Conserved Domain Database (CDsearch) [58,59]. Structural homology of the *vgrG *and *hcp *island proteins to those for which the chemical structure has been determined was performed using the HHpred server (http://toolkit.tuebingen.mpg.de/hhpred) [60,61].

## Conflicting interests

The authors declare that they have no competing interests.

## Authors' contributions

PDM, SNV, BD, TAC and THMS conceived the study. PDM, TK and THMS performed experiments and analysis. PDM, SNV, BD, TAC, and THMS wrote the original manuscript. All authors read and approved the final version.

## Supplementary Material

Additional file 1 **Table S1 Non-conserved proteins encoded in the T6SS *hcp *and *vgrG *islands in *Pantoea *and *Erwinia *species**. Table showing the Blast analysis of the proteins encoded in the *Pantoea *and *Erwinia hcp *and *vgrG *islands against the NCBI protein database [52], Conserved Domain Database using the CD-search server [58] and the Protein Database (PDB) using the Hhpred server [60,61]. For the BlastP analysis against the NCBI protein database, the highest informative Blast result outside of the *Pantoea/Erwinia *group was determined. The amino acid positions showing sequence identity, percentage amino acid (aa) identity, bit scores and BLAST E-values are shown. For the Blast analysis for conserved domains [58], the protein families and conserved orthologous groups are shown, as well as the bit scores and E-values. For the Hhpred analysis of the amino acid sequences for structural homology the Hhpred scores and E-values are shown. Click here for file
